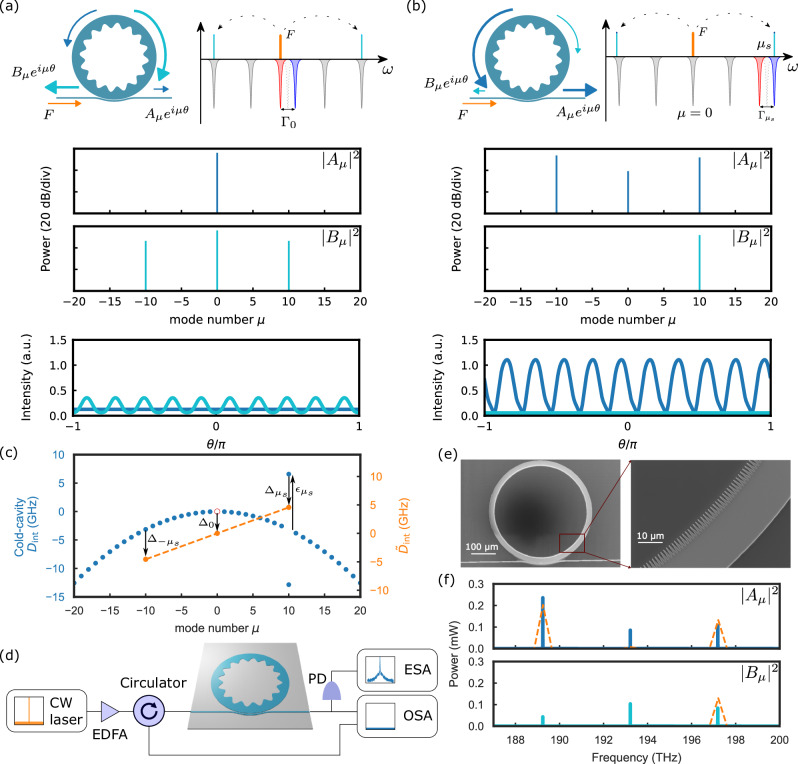# Publisher Correction: The bandgap-detuned excitation regime in photonic-crystal resonators

**DOI:** 10.1038/s41467-025-65401-y

**Published:** 2025-10-30

**Authors:** Yan Jin, Erwan Lucas, Jizhao Zang, Travis Briles, Ivan Dickson, David Carlson, Scott B. Papp

**Affiliations:** 1 Time and Frequency Division, National Institute of Standards and Technology, Boulder, CO USA; 2 Department of Physics, University of Colorado, Boulder, CO USA; 3 Laboratoire ICB, UMR 6303 CNRS-Université de Bourgogne, Dijon, France; 4 Octave Photonics, Louisville, CO USA

**Keywords:** Solitons, Nonlinear optics, Frequency combs, Nonlinear optics

Correction to: *Nature Communications* 10.1038/s41467-025-60156-y, published online 31 May 2025

In Figure 1 of this article, some ‘0’s did not display correctly; the figure should have appeared as shown below. The original article has been corrected.

Incorrect Figure

**Figure:**
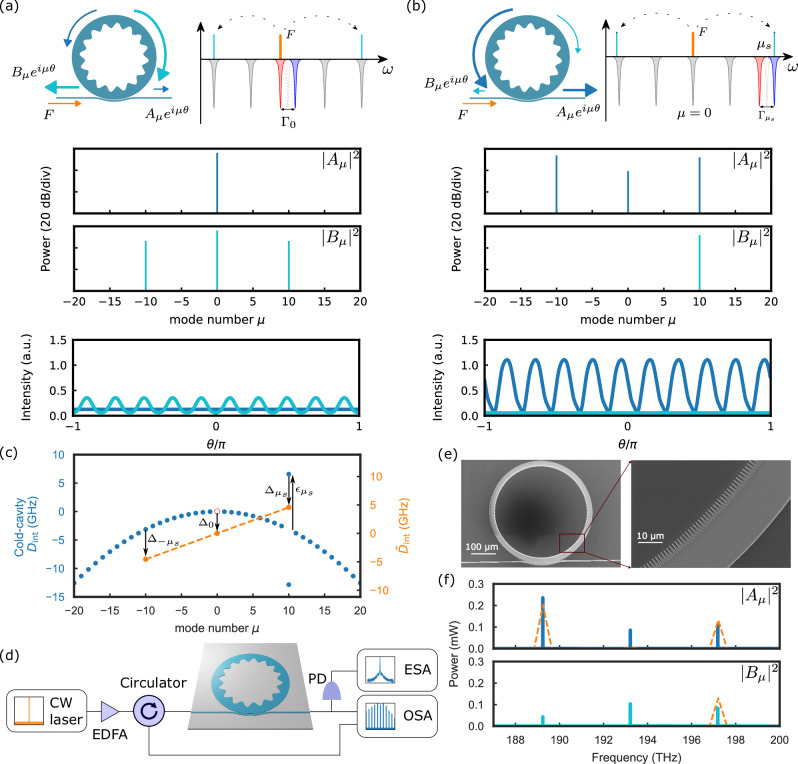

Corrected Figure

**Figure:**