# Supplementary material for: The bandgap-detuned excitation regime in photonic-crystal resonators
Source: Nat Commun. 2025 May 31;16:5077. doi: 10.1038/s41467-025-60156-y (PMC12126479; doi:10.1038/s41467-025-60156-y)
Supplement: Supplementary file 1 — Supplementary Information [file 41467_2025_60156_MOESM1_ESM.pdf]

# Supplementary Information for: The bandgap-detuned excitation regime in photonic-crystal resonators

Yan Jin*[1,2], Erwan Lucas[3], Jizhao Zang[1,2], Travis Briles[1], Ivan Dickson[1,2], David Carlson[4], and Scott B. Papp[1,2]

[1]Time and Frequency Division, National Institute of Standards and Technology, 325 Broadway MS 688, Boulder, 80305, Colorado, USA.
[2]Department of Physics, University of Colorado, 390 UCB, Boulder, 80305, Colorado, USA.
[3]Laboratoire ICB, UMR 6303 CNRS-Université de Bourgogne, 21078 Dijon, France.
[4]Octave Photonics, 325 W South Boulder Rd Suite B-1, Louisville, 80027, Colorado, USA.

Contributing authors: yan.jin@colorado.edu;

**Calculation of parametric gain** Here we present the procedure to calculate the parametric gain at the threshold. More details can be found in [1]. For the bandgap-detuned regime, we consider the OPO case with the split mode at $\mu_s$. Since there's no bandgap at the pump mode, the stationary homogeneous solution to Equations. (1) and (2) in the main text is

$$0 = -(1 + i\alpha)A_0 + iA_0|A_0|^2 + F. \tag{1}$$

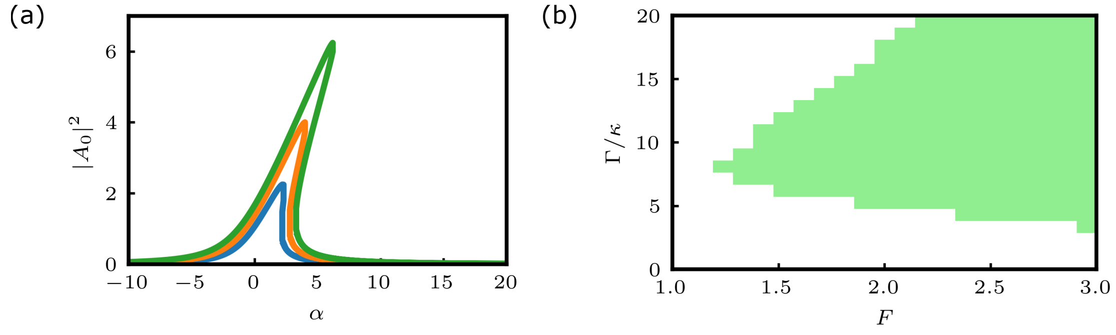


**Supplementary Figure 1** (a) Homogeneous solution in the bandgap-detuned regime. $F = 1.5$, 2 and 2.5 for blue, orange and green curves, respectively. (b) Existence map for the normalized half bandgap $\Gamma/\kappa$ and $F$.

We plot the curves of $|A_0|^2$ with respect to $\alpha$ in Supplementary Figure 1(a) for $F = 1.5$, 2 and 2.5. At the threshold, the comb lines of interest are $(A_{\mu_s}, B_{\mu_s}, A^*_{-\mu_s})$. We can write the equations in the matrix form:

$$\frac{d}{d\tau}\begin{pmatrix} A_{\mu_s} \\ B_{\mu_s} \\ A^*_{-\mu_s} \end{pmatrix} = \begin{pmatrix} -1 - iq_{\mu_s} & i\Gamma_{\mu_s}/\kappa & iA_0^2 \\ i\Gamma_{\mu_s}/\kappa & -1 - iq_{\mu_s} & 0 \\ -iA_0^{*2} & 0 & -1 + iq_{\mu_s} \end{pmatrix}\begin{pmatrix} A_{\mu_s} \\ B_{\mu_s} \\ A^*_{-\mu_s} \end{pmatrix}, \tag{2}$$

where $q_{\mu_s} = \alpha + D_2\mu_s^2/\kappa - 2|A_0|^2$.

Here we define the gain as the real part of the eigenvalues of the matrix in Equation (2). Given $D_2/\kappa$, $\mu_s$ and $F$, as we sweep the detuning $\alpha$, if the real part of any of the eigenvalues is greater than 0, then the

comb lines will be generated. The OPOs can't be generated at the region where none of the eigenvalues is greater than 0 for all $\alpha$, which corresponds to the gray area in the existence map of Figure 2(a) in the main text. As long as the eigenvalue is greater than 0, $\delta\omega_{\text{int}} = \tilde{D}_{\text{int}}(\mu_s)/\mu_s = \text{Re}\{i\dot{A}_{\mu_s}/(\mu_s A_{\mu_s})\}$ is calculated. We also plot the existence map in Supplementary Figure 1(b) for $\Gamma/\kappa$ and $F$ at $\mu_s = 10$, and the OPOs can only live within the green region. We should note that the existence map only provides the region where the gain is greater than 0, and it doesn't predict the stability or efficiency of the OPOs which need to be simulated directly by Equations (1-2) in the main text. The OPO pattern generated with reasonable $F$ and $\Gamma/\kappa$ is similar; however, with a large $F$ (usually greater than 2.5), the OPOs tend to be unstable.

We can also calculate the parametric gain for the conventional regime where the split mode is pumped ($\Gamma_\mu = \Gamma\delta_{\mu,0}$). The stationary homogeneous solutions to Equations 1 and 2 can be calculated from

$$0 = -(1+i\alpha)A_0 + i\Gamma B_0/\kappa + iA_0(|A_0|^2 + 2|B_0|^2) + F \tag{3}$$
$$0 = -(1+i\alpha)B_0 + i\Gamma A_0/\kappa + iB_0(|B_0|^2 + 2|A_0|^2), \tag{4}$$

and we provide a solution in Supplementary Figure 2(a) for $|A_0|^2$ (blue curve) and $|B_0|^2$ (orange curve) with $\Gamma/\kappa = 4$ and $F = 2.6$.

At the threshold, we calculate for un-pumped modes $\mu \neq 0$

$$\frac{d}{d\tau}\begin{pmatrix} A_\mu \\ A^*_{-\mu} \\ B_\mu \\ B^*_{-\mu} \end{pmatrix} = \begin{pmatrix} -ip_\mu - 1 & iA_0^2 & & \\ -iA_0^{*2} & ip_\mu - 1 & & \\ & & -ip_\mu - 1 & iB_0^2 \\ & & -iB_0^{*2} & ip_\mu - 1 \end{pmatrix} \begin{pmatrix} A_\mu \\ A^*_{-\mu} \\ B_\mu \\ B^*_{-\mu} \end{pmatrix}, \tag{5}$$

where $p_\mu = \alpha + D_2\mu^2/\kappa - 2(|A_0|^2 + |B_0|^2)$. Taking the values of $A_0$ and $B_0$ solved in Equations 3-4, we can find that as we increase the detuning $\alpha$, the gain for $B_\mu$ or $B^*_{-\mu}$ is greater than that for $A_\mu$ or $A^*_{-\mu}$. We plot the existence map in Supplementary Figure 2(b) and mark the region with red for the greater backward gain and with blue for the greater forward gain. This explains why pumping the split mode usually generates backward-propagating combs.

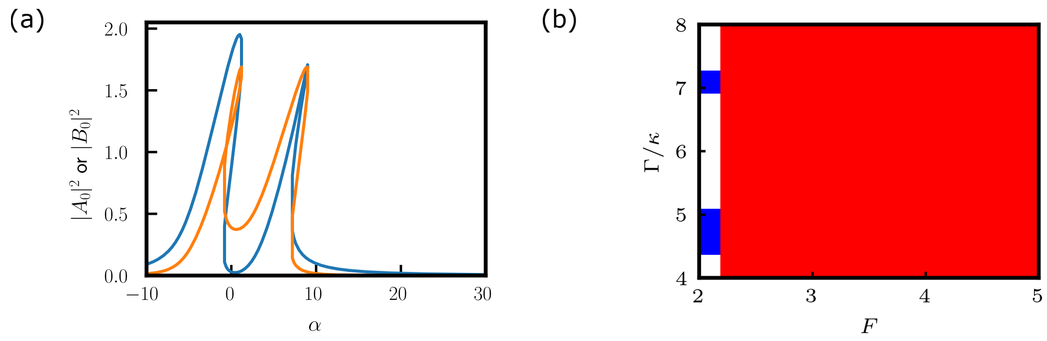


**Supplementary Figure 2** (a) Homogeneous solution for $|A_0|^2$ (blue) and $|B_0|^2$ (orange) in the conventional regime with $F = 2.6$ and $\Gamma/\kappa = 4$. (b) Existence map for the normalized half bandgap $\Gamma/\kappa$ and $F$ with given $D_2/\kappa = -0.0185$. The backward gain is greater than the forward gain in the red region, and the forward gain is greater in the blue region. The gain is never greater than 0 in the white region.

**Mode structures** Here we present the characterized mode structures for different nanostructure definition. It is clear that the split modes don't affect other unsplit modes. Supplementary Figure 3 presents the measured mode structure for the OPO cases where only 1 mode is split. The relative mode number $\mu$ and the corresponding frequency are marked for each panel. We note that as we pump different modes, the relative mode numbers including $\mu_s$ will change and the pump mode is always at $\mu = 0$. In this figure, the split mode is at $\mu_s = 0$.

Supplementary Figures 4, 5 and 6 present the measured mode structures for $\mu_s = \{-1, 1\}$, $\mu_s = \{-3, 3\}$ and $\mu_s = \{\pm1, \pm2, ..., \pm10\}$, respectively. The measured dispersion presented in the main text is derived from these mode structures.

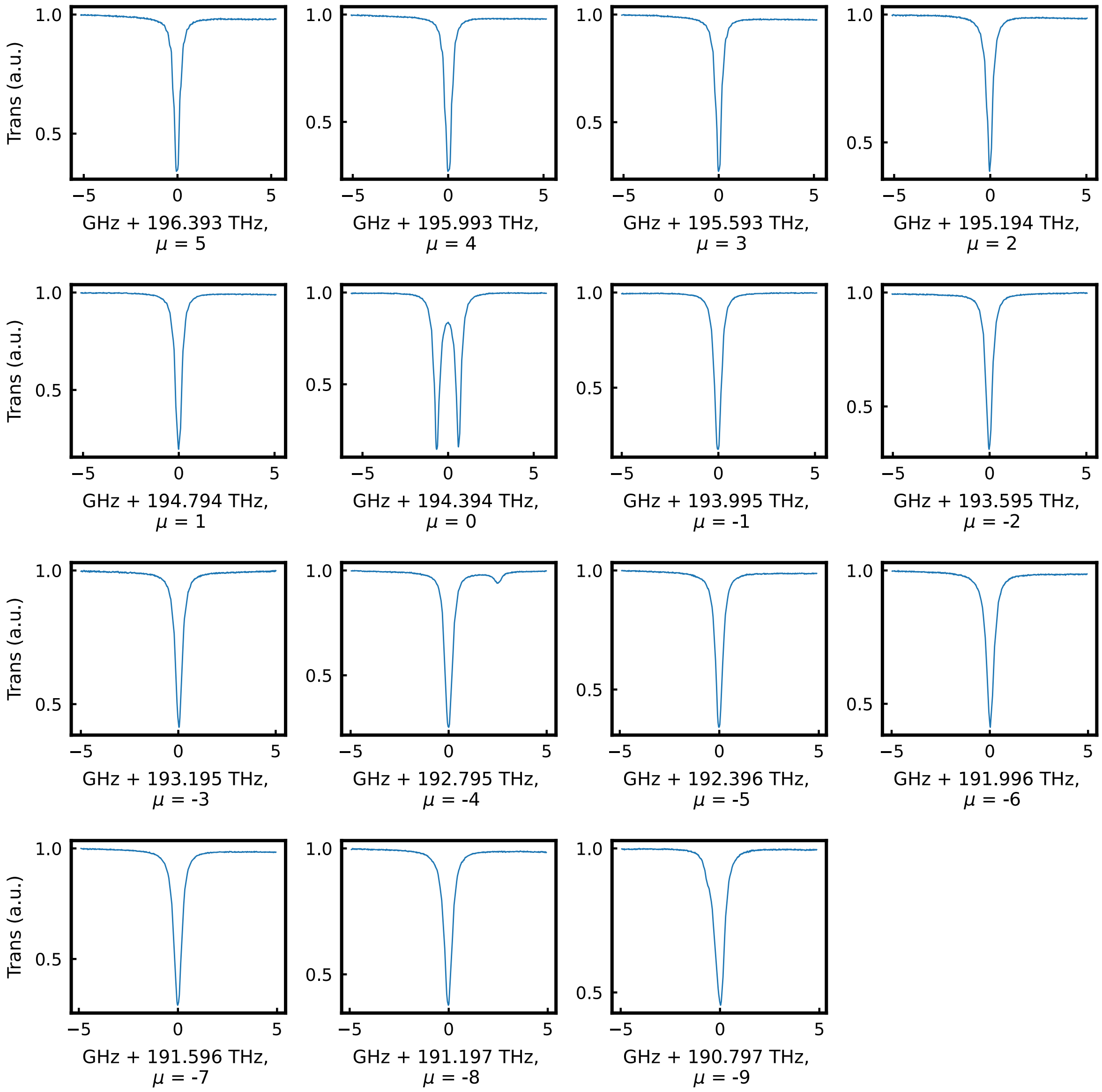


**Supplementary Figure 3** Mode structure for the ring with only 1 split mode.

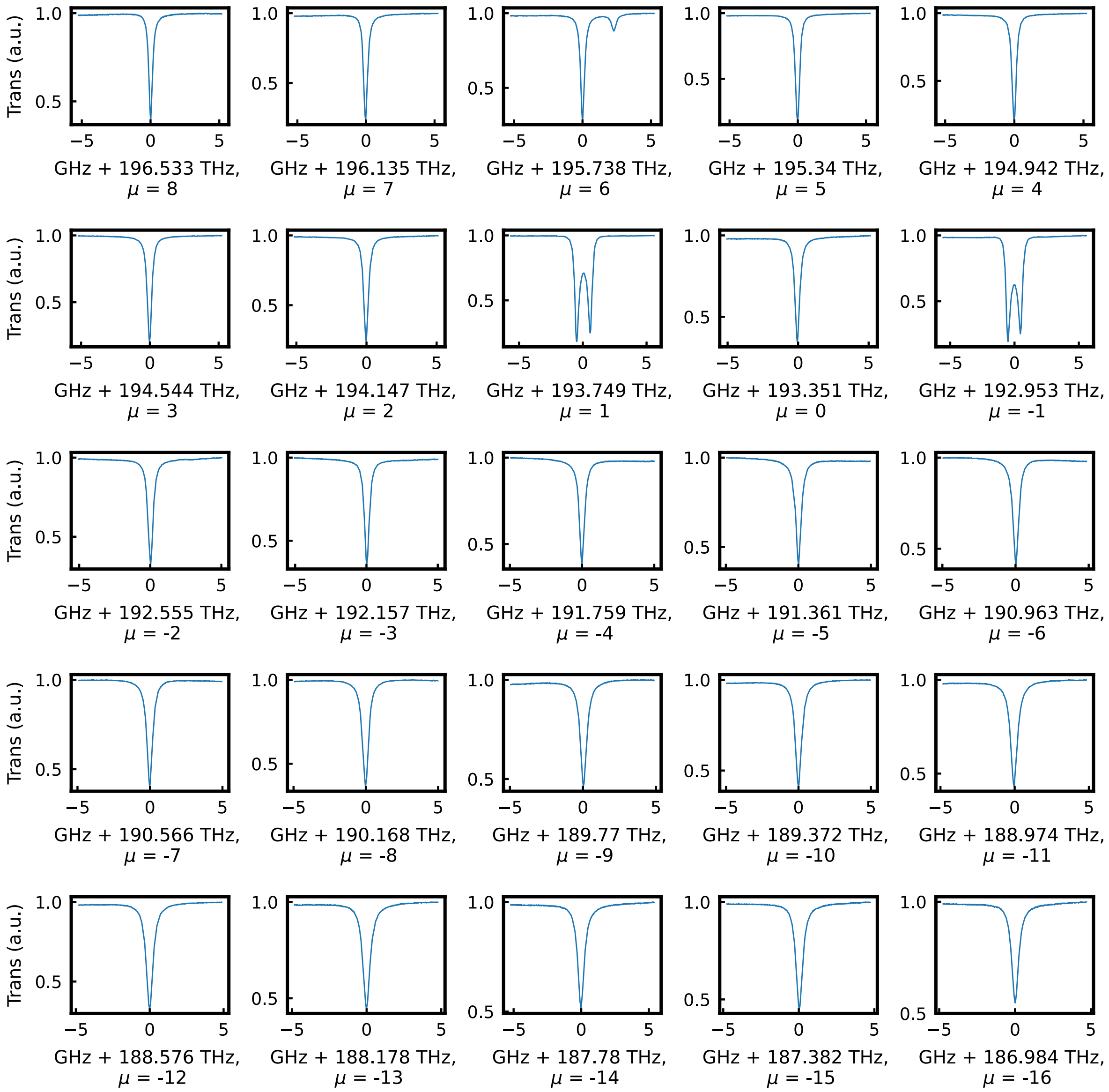


**Supplementary Figure 4** Mode structure with $\mu_s = \{-1, 1\}$ for Figure 3(f) in the main text.

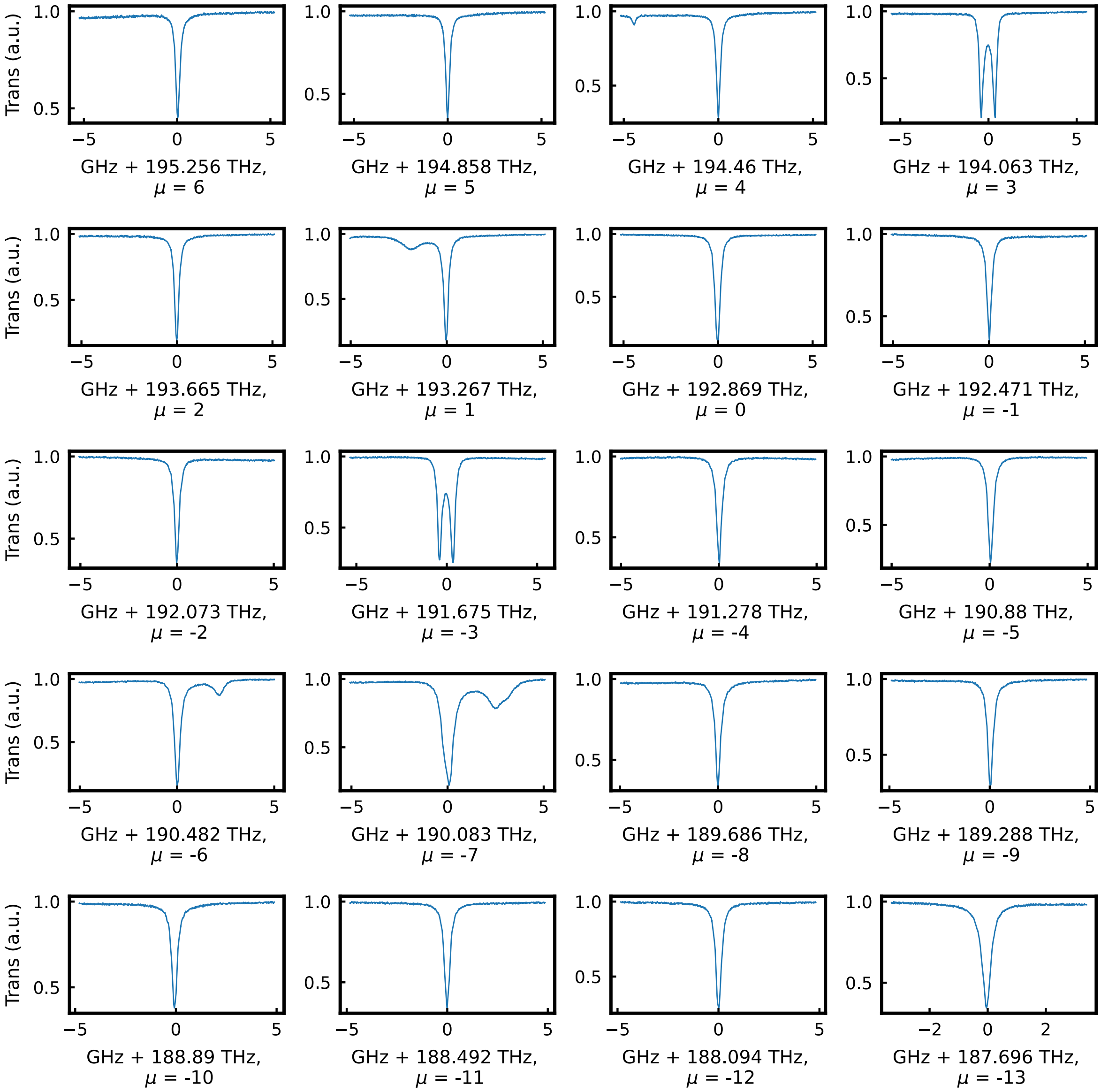


**Supplementary Figure 5** Mode structure with $\mu_s = \{-3, 3\}$ for Figure 4(a) in the main text.

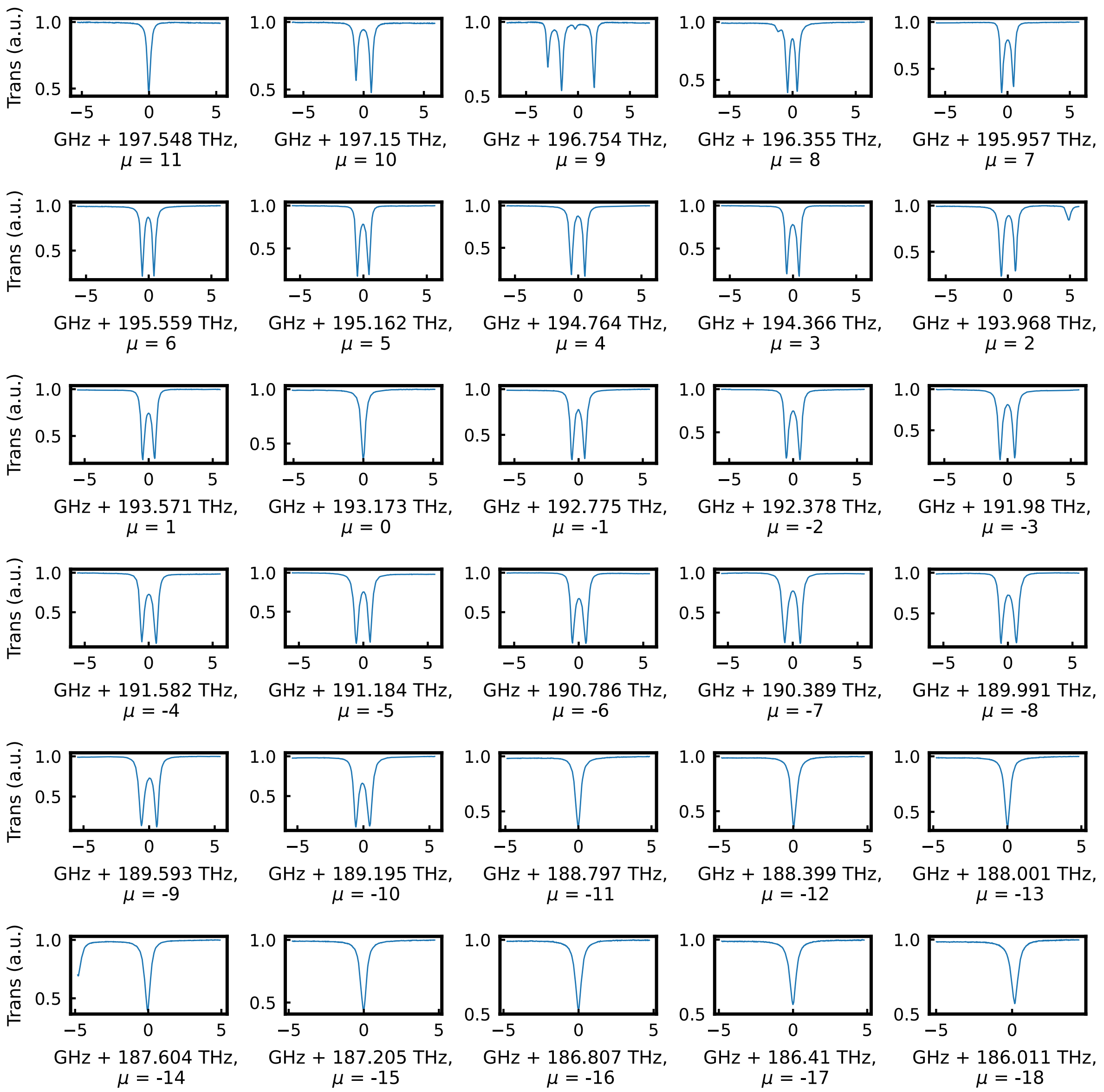


**Supplementary Figure 6** Mode structure with $\mu_s = \{\pm 1, \pm 2, ..., \pm 10\}$ for Figure 4(c) in the main text.

# References


[1] Kondratiev, N.M., Lobanov, V.E.: Modulational instability and frequency combs in whispering-gallery-mode microresonators with backscattering. Physical Review A **101**(1), 013816 (2020) https://doi.org/10.1103/PhysRevA.101.013816
